# Highly Soluble Drugs Directly Granulated by Water Dispersions of Insoluble Eudragit® Polymers as a Part of Hypromellose K100M Matrix Systems

**DOI:** 10.1155/2019/8043415

**Published:** 2019-03-05

**Authors:** Eliška Mašková, Martina Naiserová, Kateřina Kubová, Josef Mašek, Sylvie Pavloková, Martina Urbanová, Jiří Brus, Jakub Vysloužil, David Vetchý

**Affiliations:** ^1^Department of Pharmaceutics, Faculty of Pharmacy, University of Veterinary and Pharmaceutical Sciences Brno, Brno, Czech Republic; ^2^Department of Pharmacology and Immunotherapy, Veterinary Research Institute, Brno, Czech Republic; ^3^Institute of Macromolecular Chemistry, Czech Academy of Sciences, Praha, Czech Republic

## Abstract

The aim of the present study was to investigate the suitability of insoluble Eudragit® water dispersions (NE, NM, RL, and RS) for direct high-shear granulation of very soluble levetiracetam in order to decrease its* burst effect* from HPMC K100M matrices. The process characteristics, ss-NMR analysis,* in vitro* dissolution behavior, drug release mechanism and kinetics, texture profile analysis of the gel layer, and PCA analysis were explored. An application of water dispersions directly on levetiracetam was feasible only in a multistep process. All prepared formulations exhibited a 12-hour sustained release profile characterized by a reduced* burst effect* in a concentration-dependent manner. No effect on swelling extent of HPMC K100M was observed in the presence of Eudragit®. Contrary, higher rigidity of formed gel layer was observed using combination of HPMC and Eudragit®. Not only the type and concentration of Eudragit®, but also the presence of the surfactant in water dispersions played a key role in the dissolution characteristics. The dissolution profile close to zero-order kinetic was achieved from the sample containing levetiracetam directly granulated by the water dispersion of Eudragit® NE (5% of solid polymer per tablet) with a relatively high amount of surfactant nonoxynol 100 (1.5%). The initial* burst *release of drug was reduced to 8.04% in 30 min (a 64.2% decrease) while the total amount of the released drug was retained (97.02%).

## 1. Introduction

Drug delivery systems based on poly(acrylic acid) derivatives are highly suitable in order to provide time- and/or site-controlled drug delivery within the gastrointestinal tract [1–5]. Polymethacrylate copolymers (Eudragit®) are widely used either for controlled release film coatings or as matrix formers in common granulation techniques as well as in direct compression in oral pharmaceutical formulations [6–10]. These polymers appear particularly attractive due to their high chemical stability, good compatibility properties, and a large variety of products with different physicochemical characteristics present on the market [11–13].

Polymethacrylates are synthetic cationic, anionic and neutral polymers of dimethylaminoethyl methacrylates, methacrylic acid, and methacrylic acid esters in varying ratios. Their physicochemical properties are determined by functional groups. Several different types are commercially available and they can be obtained as dry powder, granules, an aqueous dispersion, or an organic solution [6, 9, 14]. 

The insoluble types of polymers Eudragit® RL and RS with alkaline as well as Eudragit® NE and NM polymers with neutral groups enable controlled time release of the active ingredient by pH-independent swelling [9, 14]. They are used in sustained release drug delivery separately or in combination with other poly(meth)acrylates (pH-dependent soluble or insoluble type of Eudragit®) [12, 15] or with differently structured polymers, very often with cellulose derivatives such as hypromellose (HPMC) [16, 17].

The hydrophilic polymer selected for the present study was hypromellose K100M (HPMC K100M). Hydrophilic polymer matrix systems are widely used for designing oral sustained release delivery systems because of their flexibility to provide the desirable drug release profile, cost effectiveness, and broad regulatory acceptance [16]. The drug release from HPMC polymer matrix, especially the release of highly water-soluble drugs formulated into HPMC matrices, may be characterized by an initial* burst effect,* because of the rapid diffusion of the dissolved drug through the hydrophilic gel network created on the matrix surface. The initial high release rate may lead to drug concentration near or above the toxic level* in vivo* [18].

A combination of water-insoluble polymers with HPMC could solve this problem as they decrease the penetration of water in the matrix, leading to a decreased diffusion of the drug and slower initial release. The insoluble polymers that could be incorporated into the HPMC matrices include insoluble methacrylic acid copolymers (e.g., Eudragit® NE 30D) and ammonio-methacrylate copolymers (e.g., Eudragit® RL 100 or PO RS 100, RS 30D), ethylcellulose (e.g., Ethocel® or Surelease®), cellulose acetate, or polyvinyl acetate [9, 19–24]. To reduce the* burst effect,* an ethanolic solution of Eudragit® RL 100 and RS 100 was added to a mixture of freely water-soluble drug nicorandil/HPMC K4M [25].

As a more effective tool proved to be the application of an insoluble polymer in a liquid form directly on the API (Active Pharmaceutical Ingredient) or API/insoluble filler mixture, currently, the information on this issue is relatively limited in the literature and available only for non-HPMC matrix systems. In the study published by Krajacic et al., the slightly soluble diclofenac sodium was successfully granulated with the aqueous polymer dispersion of Eudragit® NE 40D to obtain prolonged drug dissolution profiles from matrices without the burst release [26]. Similarly, Tabasi et al. [27] described the granulation of the mixture of slightly soluble theophylline/lactose monohydrate/microcrystalline cellulose (PH 101) with a different concentration of Eudragit® NE 30D to investigate the dissolution behavior of prepared matrices. On the other hand,* burst effect* of freely soluble drug diltiazem hydrochloride was reduced by applying Eudragit® NM 30D on the drug/insoluble microcrystalline cellulose (PH 101) blend in the ratio 1:1 during the high-shear granulation [28]. Additionally, Tsai et al. [29] granulated conventional excipients, such as lactose and dibasic calcium phosphate with the Eudragit® RS 30D, Eudragit® RL 30D and ethylcellulose (Surelease®). Granulated excipients were directly compressed to fabricate sustained release captopril tablets.

In our previous recently published studies focused on HPMC K4M matrices with a reduced* burst effect*, we investigated an application of water dispersions of insoluble Eudragit® polymers on a very soluble model API during high-shear granulation. It was concluded that there is no possibility of applying them directly in one-step process due to the creation of a highly sticky mass unsuitable for consequent processing. This problem was solved by the addition of insoluble filler such as MCC or Neusilin® US2 to create a drug/filler mixture [30, 31]. Nevertheless, the behavior of the highly soluble API directly granulated with water dispersions of insoluble Eudragit® polymers has not been published yet. The main aim of the present study was to explore a near zero-order release of highly soluble model drug directly granulated by water dispersions of Eudragit® NE, NM, RL, and RS, respectively, from hypromellose K100M matrix tablets. Moreover, the matrix systems were investigated using gel layer investigation and multivariate data analysis. The integral part of this study was a presentation of difficulties observed during the granulation process.

## 2. Materials and Methods

### 2.1. Materials

Levetiracetam was kindly donated by Zentiva k.s., Prague, Czech Republic. HPMC K100M was procured from Colorcon Limited, Dartford, Great Britain, and the microcrystalline cellulose (MCC) Avicel® PH 102 was purchased from FMC Biopolymers, Rockland, United States of America. Eudragit® NE, NM, RL, and RS in the form of 30% water dispersions were received as a gift sample from Evonik Röhm GmbH, Darmstadt, Germany. Magnesium stearate was obtained from Peter Greven, Bad Műnstereifel, Germany and colloid silica from Degussa, Vicenza, Italy. All materials were of European Pharmacopoeia (Ph. Eur.) quality.

### 2.2. Methods

#### 2.2.1. Preparation and Evaluation of Granules

The granules from the model drug levetiracetam (particle size 141.57 ± 28.7 *μ*m, laser diffraction HELOS, Sympatec, Germany, aqueous solubility at 20°C 104 g/100 ml [32]) and 30% aqueous dispersion of four different insoluble Eudragit® polymers were prepared in a high-shear mixer (ROTOLAB, Zanchetta, S. Salvatore Montecarlo, Italy). The instrument settings were as follows: impeller pause time, 0 s, impeller working time, 300 s, cycle time, 300 s, and impeller speed, 1200 rpm. The granulation liquid (30% water dispersion of Eudragit® polymers) was manually added for the first 60 seconds, then the mixture was granulated for 240 s at 1200 rpm. The wet mass was passed through a 1.25 mm mesh sieve and the granules were dried for 24 hours at 40°C in a cabinet dryer. After drying, the granules were again passed through the 1.25 mm mesh sieve.

The amount of water dispersion used in the granulation process was limited by the absorption capacity of the drug, which allowed an application of only a limited amount of a 30% Eudragit® water dispersion on the drug in individual steps of the granulation process. Set 2.5 including samples NE2.5, NM2.5, RL2.5, and RS2.5 was prepared by the application of 13.9, 6.9, and 7.0 g of water dispersion in three-step granulation. Set 5 including samples NE5, NM5, RL5, and RS5 was prepared by the application of 13.9, 6.9, 7.0, 8.8, 10.0, and 9.0 g in a six-step granulation process. The final granule composition is shown in Table 1. This multistep granulation helped to avoid overwetting of the granulated mass.

Three samples of approximately 1 g were taken from dry granules after last granulation step and they were evaluated for their humidity. Each sample was put on the hanging pan of Moisture Analyzer HX 204 (Mettler-Toledo AG, Switzerland) and it was dried for 15 minutes at 105°C by a halogen radiator. Sample weight was recorded continually by integrated balance. The total loss in weight was interpreted as moisture content.

The other excipients, the hydrophilic polymer HPMC K100M, the indifferent insoluble filler Avicel® PH 102, magnesium stearate (2.5%) and colloid silica (0.5%), both for improving granule flow properties, were added extragranularly and the mixing procedure continued by a 3-axial homogenizer Turbula (T2C WAB, Basel, Switzerland) for another 10 min.

The higher concentration of magnesium stearate than usual (2.5%) was chosen to improve flowability of tableting mixtures, otherwise exhibiting poor flow characteristics without magnesium stearate. The same concentration was used also in other experimental studies [33, 34]. Considering the excellent mechanical properties of matrix tablets (see later), an often described weakening of the particle-particle bonding was not observed. No effect of magnesium stearate on slow-down of dissolution profile was expected because the particles of levetiracetam were not in direct contact with it in the matrix structure.

The prepared granules were evaluated according to Ph. Eur. 9 for flowability (Medipo, Brno, Czech Republic; diameter of outflow opening 25.0 ± 0.01 mm), Hausner ratio (SVM 102, Erweka, Heusenstammen, Germany).

#### 2.2.2. Solid-State NMR Spectroscopy

The ^13^C cross-polarization (CP) magic angle spinning (MAS) NMR spectra were measured at a spinning frequency of 11 kHz, a B_1_(^13^C) field nutation frequency of 62.5 kHz, and contact time of 1 ms and with the repetition delay of 7 s. The ^13^C-detected* T*_1_(^1^H) relaxation times were measured using a saturation-recovery experiment in which the initial train of ^1^H saturation pulses was followed by a variable delay (0.01–15 s). The intensity of the ^1^H spin-locking field in the frequency units was 80 kHz. Glycine was used as an external standard to calibrate the ^13^C NMR chemical shift scales (176.03 ppm), respectively. The experiments were conducted at 295 K. Frictional heating of the sample was compensated and the sample temperature was calibrated using ^207^Pb chemical shift in Pb(NO_3_)_2_ [35].

#### 2.2.3. Preparation and Evaluation of Matrix Tablets

Hydrophilic matrix tablets of approximate weight 340 mg were compressed using 10 mm-diameter lentiform-faced punches (Korsch, type EK 0, Korsch Pressen, Berlin, Germany). The compaction force was 21.5 kN. The composition of the matrix tablets is shown in Table 2. The reference sample (R) containing polymer MCC PH 102 instead the Eudragit® was prepared by direct compression.

All tablets were evaluated according to Ph. Eur. 9 for weight uniformity (n = 20, analytical balance KERN 870-13, KERN & Sohn Gmgh, Germany), hardness (n = 10, C50 Tablet Hardness & Compression Tester, Engineering Systems, Nottingham, Great Britain) and friability (n = 1, weight of 6.5 g at 25 rpm for 4 min, TAR 10, Erweka, Heusenstamm, TAR 10, Germany). An YL 9100 high-performance liquid chromatograph (HPLC, Young Lin Instrument) was used to determine the levetiracetam content (n=10). Chromatographic separation was implemented in column Venusil XBP C18 (150 × 4.6 mm; particle size 3 *μ*m). The mobile phase contained 68% of 0.2% phosphoric acid and 32% methanol. In total, the analysis took 5 minutes and the chromatograms were detected at 220 nm. The mobile phase flow rate was 1.0 ml/min, column temperature 27°C and injection volume was 20 *μ*l. To confirm the stability of levetiracetam in matrix tablets, the evaluation of the content was repeated again after 12 months (condition 25°C, 60% of relative humidity) (data not shown).

#### 2.2.4. Determining Dissolution Profiles and Similarity Factor Analysis

The dissolution profiles of the prepared samples were determined by a 12-hours dissolution test with SOTAX AT 7 On-Line System (Donau Lab, Zurich, Switzerland) using the paddle method at 50 rpm in 900 ml of a phosphate buffer (pH 6.0, Ph. Eur. 9) at 37°C. The samples were withdrawn at times 30 min, 60 min and then each 1 hour, and quantified for the released drug amount by HPLC according to the above-mentioned conditions. The mean value of the drug release (n = 6) and the standard deviation (SD) for each tablet batch were calculated.

In order to compare the dissolution profiles of levetiracetam, similarity factors* f*_*2*_ were calculated. The similarity factors were determined between the samples to examine the effect of the Eudragit® type and concentration on the dissolution profiles of the model drug. The data were analyzed by (1) for similarity factor* f*_*2*_ [36, 37]:

(1) $$\begin{array}{c} f_{2}=50\times \log\left\{\;\left.\;{\left[\;1+\left(\frac{1}{n}\right)\sum_{i=1}^{n}{\left|R_{i}-T_{i}\right|}^{2}\;\right]}^{-0,5}\times 100\;\right\}\;\right. \end{array}$$

where* n* is the number of time points;* R*_*i*_ and* T*_*i*_ are the dissolution data of reference and tested samples at time* i*. The similarity factor value ranges between 0 and 100. When the similarity factor is equal to 100, the two profiles are identical. When it approaches 0, their dissimilarity increases. Values between 0 and 50 express a statistically significant difference in dissolution curves. On the other hand, values ranging from 50 to 100 confirm statistically significant similarity [37].

#### 2.2.5. Drug Release Kinetics and Mechanism Analysis

The mechanism and kinetics of drug release from matrix tablets were studied by correlating the obtained dissolution data with the following equations: square root-time kinetics (zero-order equation, first-order equation, Higuchi model, Korsmeyer-Peppas equation, Hixson-Crowell model and the Baker-Lonsdale model ([37–41]).

#### 2.2.6. Determination of Gel Layer Thickness and Penetration Force

The test consisted of each tablet being placed into a holder made of polyvinylchloride (PVC) which covered the tablet, and allowed the polymer to swell in only one direction. The gel layer thickness of the swelled matrices and penetration force, depicted by work performed as a function of time, were measured every hour under the same conditions as the dissolution test in an* off-line* apparatus using a Texture Analyser CT 3 (Brookfield, Great Britain) for 6 hours [42].

After being pulled out of the dissolution vessel, the tablet in the PVC mold was placed in the center of the testing platform. A TA39 cylinder/rod probe (2 mm in diameter and 30 mm depth) was used to determine these parameters. The probe was moved towards the sample at a speed of 0.5 mm/s. The measurement of the penetration depth and penetration force was started when a trigger load of 5 g was achieved (which corresponded to the contact of the probe with the edge of the gel layer). The measurement was terminated when a trigger load of 250 g was reached (corresponding to the contact of the probe with a border between the hydrated gel layer and nonhydrated polymer in the core of matrix). Then the probe was automatically withdrawn from the gel layer at a speed of 10 mm/s [43]. The obtained data were analyzed at a rate of 200 points per second using Texture Expert software (Brookfield, Great Britain). The mean value of the three samples and SD for both parameters were calculated for each individual time point.

#### 2.2.7. Characterization of Gel Layer by Cryo-Scanning Electron Microscopy (Cryo-SEM)

The surface morphology of the matrix tablet NM2.5 was observed using a scanning electron microscope Hitachi SU8010, Japan. The matrix tablets were allowed to swell in a dissolution medium under the same conditions as the dissolution test. A special ceramic tablet-shaped container was used to restrict tablet swelling to one direction, the top surface of the tablet. Tested tablets were removed from the vessels, cross-sectioned and immediately frozen in liquid nitrogen after 3 hours. Finally, the frozen cross-sections were coated with Pt/Pd and the structure and the magnitude of the gel layer were observed on the cross-sections at -130°C.

#### 2.2.8. Multivariate Data Analysis

When comparing the effects of a sample composition on selected variables, an analysis of variance (ANOVA) was employed to determine the statistical significance (significant effect for cases where the p-value is less than the level of significance, *α* = 0.05, and insignificance for p-value greater than 0.05). The p-values are listed in parentheses for commented parameters in the Discussion section.

For visualization of the data structure and dependencies among the variables, a PCA was carried out after the data standardization. Especially the sample comparison on the basis of polymer type (reference, Eudragit® samples) and Eudragit® concentration was mainly examined. The resulting model was built on the grounds of selected variables providing a good correlation structure and explained variability. A total of 7 variables were included in the analysis: tablet hardness, release exponent* n* for the Korsmeyer-Peppas kinetic model designated as* n (KPM), *determination coefficient for zero-order kinetics* R*^*2*^* (0)*, and the amount of released drug in dissolution time 30 min (*burst effect*), 300 min (the time point when the penetration force including to PCA was measured), and 720 min (the final time of the dissolution test); from the dataset, time of 300 minutes was selected based on the most proven significant effects in ANOVA outputs.

The data analysis was performed with the aid of software R, version R 3.4.3 [44].

## 3. Results and Discussion

### 3.1. Preparation and Flow Properties of the Granules

The granules were prepared by the application of Eudragit® NE, NM, RL, or RS 30% water dispersions (WD) as granulation liquids directly on the very soluble model drug levetiracetam in a high-shear mixer (Table 1). The addition of WD in one step was not achievable. Huge overwetting due to API solubility and creation of strongly sticky mass unsuitable for other use was observed [45]. Therefore, the granules of Set 2.5 and Set 5 were made by a 3-step and a 6-step process, respectively. The procedure used proved to be a time- and energy-consuming process. Compared to our previous results, the levetiracetam/microcrystalline cellulose mixture (100g : 50g) was granulated under the same conditions by 27.7 g of all types of Eudragit® WD in 1-step and by 55.7 g of Eudragit® NE or NM in 1-step and RL or RS in 2-step process [30]. Even more Eudragit® NE WD (111.3 g) was applied on the levetiracetam/Neusilin® US2 mixture (100g : 100g) in our experimental study published by Naiserova et al. [31]. Thus a conclusion can be clearly made that highly soluble API cannot be directly granulated by WD of insoluble Eudragit® polymers in any technologically tolerable process. The reduction in the number of granulation steps was achieved by applying Eudragit® WDs on the mixture of an API with an excipient exhibiting high water absorption capacity and specific surface area in a manner dependent on its amount [46, 47].

The moisture content in the dry granules after the last granulation step ranged from 0.58 to 0.93% with maximum SD 0.24%, which proves that drying was complete. Generally, a moisture content of less than 2% indicates optimum drying of granules [48].

The final prepared granules were characteristic of their flow properties according to Ph. Eur. 9 for Hausner ratio (HR) (Table 3). The Hausner ratio for the reference sample R was 1.32; for Eudragit® samples it reached values from 1.22 to 1.30 while the flow characteristic of the granules varied from fair to passable. An improvement in the granule flow properties (HR) was observed after the addition of a higher Eudragit® amount (compare Set 5 to Set 2.5). The higher proportion of a binder used for the granulation process leads expectedly to an enlargement of the granule particle diameter resulting in better flow behavior [49].

### 3.2. Solid-State NMR Spectroscopy of the Granules

The successful development and application of pharmaceutical solid dispersions (multicomponent formulations) also require precise structural and physicochemical characterization as any structural deviation from the expected architecture may induce undesired changes in physicochemical properties. Generally, the structural transformations as well as the reduced size of crystallites or other domains have a substantial impact on the system behavior. Therefore, studying polymer-drug interactions in solid dispersions and relating the chemical composition, 3D architecture, and physicochemical properties to one another is a fundamental and challenging step in the development of these pharmaceutical solids. In this regard, solid-state NMR spectroscopy has already proven its remarkable potential.

**Figure 1:**
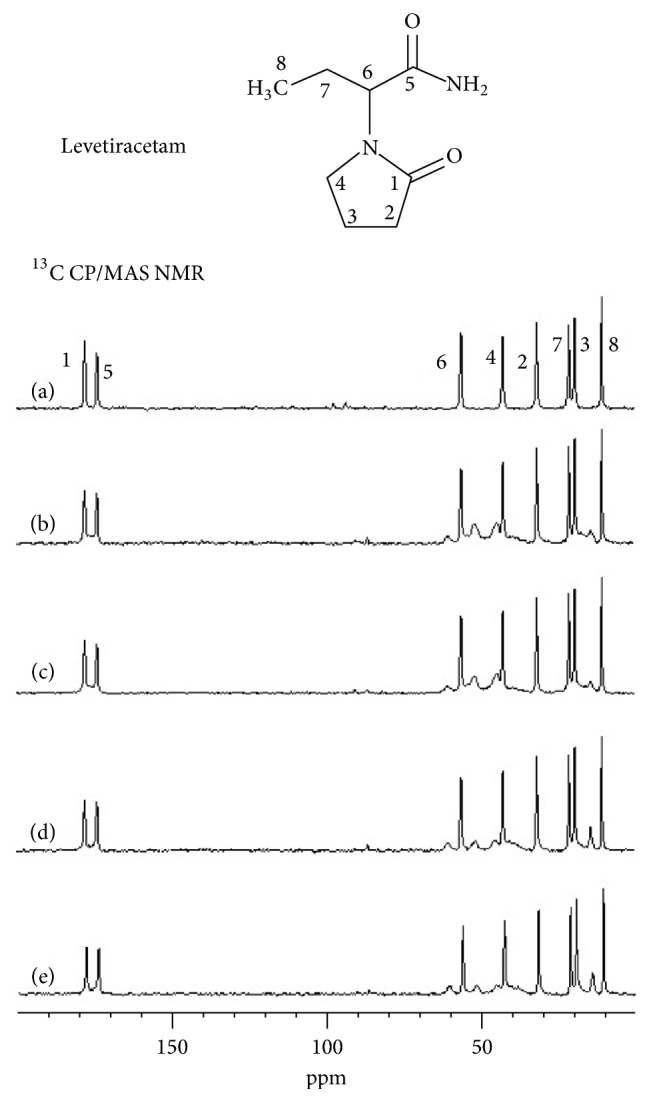
^13^C CP/MAS NMR spectra of (a) the neat levetiracetam; (b) granules levetiracetam with Eudragit® RS; (c) levetiracetam with Eudragit® RL; (d) levetiracetam with Eudragit® NM; (e) levetiracetam with Eudragit® NE.

Following our previous structural investigations of a range of pharmaceutical systems, the most suitable techniques of ss-NMR spectroscopy were applied to probe changes in the physical state and size of domains of levetiracetam depending on the presence of different polymer conformers (Eudragit® NE, NM, RL or RS). In accordance with our previous study [31], the recorded ^13^C CP/MAS NMR spectra of all the prepared granulate samples (Figure 1) clearly confirmed the structural and physicochemical stability of levetiracetam. As the recorded ^13^C CP/MAS NMR signals of levetiracetam are narrow and well separated with constant resonance frequencies regardless of the type of Eudragit® used, the active compound in the prepared solids dispersions (formulations) remains in the crystalline state and does not exhibit any phase transformations or significant increase in structural defects.

Furthermore, to estimate the size of domains of levetiracetam in the investigated granules we used the previously described experimental approach based on the measurement of ^13^C-detected ^1^H spin-lattice relaxation times (*T*_1_(^1^H)). As described previously, the ^1^H−^1^H spin diffusion, a fast magnetization transfer over large distances taking place during the relaxation periods, induces the equilibration of ^1^H magnetization behavior of different nuclear spins representing different molecules or components. To put it bluntly, if the recorded* T*_1_(^1^H) relaxation times of different components in a multicomponent system differ considerably, then the system is phase-separated with large domains the size of which usually exceeds 100-500 nm. On the contrary, if the recorded* T*_1_(^1^H) relaxation times of these components are similar or identical, it means that the system is rather homogenous with the size of domains smaller than 10 nm [50–53]. In general, the rate of magnetization equilibration reflects the extent of phase separation in multicomponent and multiphase systems.

In our particular case, the* T*_1_(^1^H) relaxation times determined for neat levetiracetam (39 s) and Eudragit® samples (2 s) differ considerably. This finding thus allows the application of the above-described experimental approach to probe changes in the size of domains. Thus, subsequently we recorded the ^13^C-detected ^1^H spin-lattice relaxation times for both components (levetiracetam and Eudragit®) for all the prepared solid dispersions. We found out that* T*_1_(^1^H) relaxation times of levetiracetam slightly decreased to the values 35, 35, 27 and 30 s for the systems with Eudragit® NE5; NM5; RL5 and RS5, respectively. This finding indicates that the size of crystallites of levetiracetam is basically unaffected (or slightly reduced) by the presence of low amounts (ca. 10 %) of Eudragit irrespective of the type of the polymer used. In contrast, as demonstrated in our previous contribution [31], higher amounts of polymer additives (ca. 25 %) induce a considerable reduction in the size of domains as well as of levetiracetam depending on of content Eudragit® NE. Due to the very high solubility of the levetiracetam and a high amount of HPMC K100M in final tablets, the change in particle size can be considered as insignificant [54].

### 3.3. Characteristics of Matrix Tablets

The results of friability, hardness, average drug content, and average weight of prepared matrix tablets are provided in Table 3. The results show that all physical parameters of the compressed tablets were within the permissible limits of Ph. Eur. The average weight of the samples was within the range of 338.7-347.2 mg. The drug content ranged from 95.95 to 106.56% and the maximum change in the content of samples after 12 months of 2.32% confirmed the stability of API in the system (data not shown). The matrices were pressed to the maximum hardness. Based on the hardness value of the reference sample R (81.6 N), it is clear that the presence of Eudragit® polymer in the structure of the tablets led to an increase in this parameter (86.3–96.8 N). This effect was evaluated as statistically significant (ANOVA, p < 0.001). In general, the addition of Eudragit® polymers improved bonding among particles ([28, 55]). After the exclusion of the reference sample from the analysis, both parameters—the type of Eudragit® used (p < 0.001) and its concentration (p = 0.002)—as well as the interactions between them (p < 0.001) had a statistically significant effect. The friability varied between 0.06% and 0.38%.

### 3.4. Dissolution Profiles, Similarity Factor Analysis, Drug Release Mechanism, and Multivariate Data Analysis

**Figure 2:**
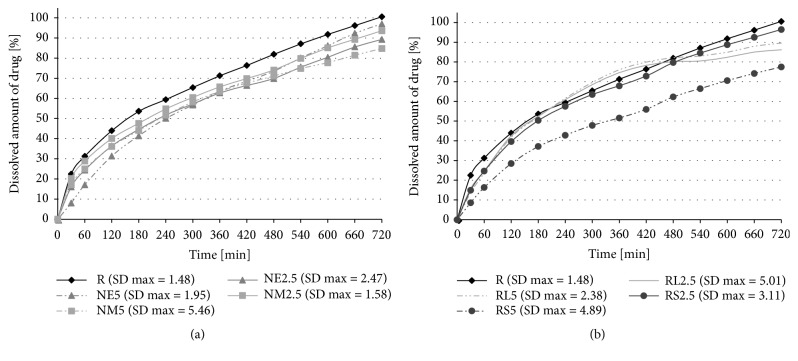
Release amount of levetiracetam from (a) formulation NM and NE and (b) formulation RL and RS during the dissolution tests in comparison with the reference sample (R) at pH 6.0.

The main aim of this study was to adjust the 12-hour dissolution profile of very soluble model drug levetiracetam from HPMC K100M matrices towards zero-order kinetics and to decrease the* burst effect* by applying the WD of insoluble Eudragit® polymers directly to the API during the granulation process. The dissolution characteristics, similarity factor analysis results, and the fittings of API release data to different kinetic equations can be seen in Figure 2, Tables 4 and 5, respectively.

**Figure 3:**
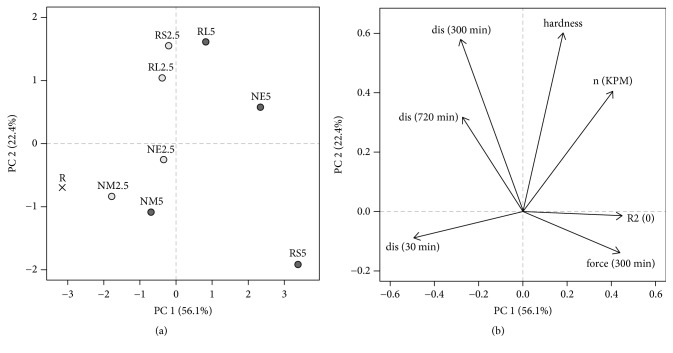
PCA scores and loadings plot: (a) PCA scores plot: objects included in model: reference sample R and Eudragit® samples; (b) PCA loadings plot: variables included in model: hardness, release exponent n for the Korsmeyer-Peppas kinetic model n (KPM), coefficient of determination for zero-order kinetics R^2^ (0), the amount of the released drug at time 30 min (burst effect), dissolution time 300 min and 720 min, and penetration force for time point 300 min.

The calculated PCA model was created for a comparison of the reference sample R and samples with a different type and concentration of Eudragit® on the basis of fundamentally important characteristics (tablet hardness, drug release mechanism expressed as a release exponent* n* from Korsmeyer-Peppas kinetic model, release kinetics expressed as a determination coefficient R^2^ for zero-order kinetics, dissolution characteristics in 30, 300 and 720 minutes, and penetration force in 300 minutes), while maintaining good correlation structure of data. Obtained outputs are represented in the PCA scores plot (Figure 3(a)) and PCA loadings plot (Figure 3(b)). The amount of variability explained by the first two principal components was high enough since it described 78.5% of variability [56].

The PCA scores plot (Figure 3(a)) shows a dissimilarity between the reference sample R and Eudragit® samples. Compared with the reference sample R, a 10%-64.2% decrease in the burst release (see* arrow dis(30 min)* in Figure 3) and a 3.5%-23% decrease in the total amount of levetiracem released from Eudragit®/HMPC matrices during the 12-hour dissolution test were observed. The influence of Eudragit® incorporated into the matrix system is attributed to its insoluble character limiting the penetration of the dissolution medium [57, 58] or to the creation of nonuniform polymer layers on the API surface leading to a decrease in the surface area for drug release [59]. Despite the high correlation with first-order kinetics (R^2^ > 0.985), all Eudragit® samples exhibited an improvement in fitting to zero-order kinetics (R^2^ > 0.912) in comparison to the reference sample R (R^2^ = 0.902); see* arrow R*^*2*^*(ZO) * in Figure 3.

While the increasing Eudragit® concentration in matrices always led to a further reduction in the* burst effect*, the results dealing with the total amount of the released drug were less unambiguous. With a rising Eudragit® concentration, an undesired decrease in the total amount of the released drug was revealed in the NM and RS samples, and this phenomenon makes them unsuitable for the desired purpose. Although the dissolution profile of the sample RS5 exhibited a very low* burst effect* (8.61%), a total deflection of the curve from R can be observed (similarity factor* f*_*2*_ R/RS5, 44.05). An overall slow-down of the dissolution profile of the RS5 sample (*T*_50%_ 337.2 ± 15.4 min) exhibiting high correlation with zero-order kinetics (R^2^ = 0.947, see Figure 3,* R*^*2*^*(0)*) was probably caused by the low permeability of this cationic polymer containing only 5% (w/w) of trimethylammonio ethyl methacrylate chloride with hydrophilic quaternary ammonium groups [60]. On the other hand, the sample NM5, with the smallest reduction of the* burst effect* (17.18%) among the samples containing 5% of insoluble polymer, exhibited a significantly lower release rate of the drug in the second half of the dissolution test (similarity factor* f*_*2*_ R/NM5 61.00). Conversely, a desired increase in the final amount of the released drug towards the reference sample R was revealed for the NE and RL samples while the Eudragit® concentration in matrices increased. Due to its higher permeability (10% w/w of trimethylammonio ethyl methacrylate [60], the sample RL5 did not exhibit a satisfactory reduction in the* burst effect* at the beginning of the dissolution test (13.70%). Different behavior was recorded for the NE5 sample. The incorporation of 5% of Eudragit® NE resulted in the dissolution profile characterized by the low burst release of very soluble levetiracetam (8.04%) and its almost complete liberation from the matrix system during 12 hours (97.02%). Therefore, this formulation was more beneficial over the sample RS5 which exhibited similarly low* burst effect* but a significantly lower total drug release. Moreover, the determination coefficient R^2^ of sample NE5 for zero-order kinetics (R^2^ = 0.958) calculated from these data was the closest to 1. Although the obtained dissolution profile was similar to the reference sample R (similarity factor* f*_*2*_ R/NM5 52.55), its behavior can be concluded as the best fitting with the desired purpose. It can be noticed that the samples based on chemically identical polymer Eudragit® NE and NM (both defined as low permeable) [6] exhibited a significantly different manner in drug release (see Figure 3(a). PCA scores plot,* arrow, dis 30 min*). This could be probably explained by the difference in the nature and content of the emulsifier. While Eudragit® NE 30 D contains nonoxynol 100 in the concentration of 1.5% (w/v), Eudragit® NM 30 D contains polyethylene glycol stearyl ether in less than a half of the concentration [6]. The presence of surfactant nonoxynol 100 in the concentration of 1.5% could probably increase the low permeability of Eudragit® NE polymeric film inside the matrix and effectively facilitate the medium penetration not at the beginning but in the next course of the dissolution test [61]. For completeness of information it should be noted that WD of Eudragit® RL and RS are surfactant-free [14].

The mechanism of API release from HPMC K100M matrices was investigated based on mathematical modelling (Table 5) and PCA results (Figures 3(a) and 3(b),* n(KPM)*). The dissolution data showed a good agreement with the Korsmeyer-Peppas model (R^2^ > 0.984), therefore the release exponent* n* could be used to predict the mechanism of the drug release. For sample, R without Eudragit® polymer,* n,* was calculated to be 0.4751. This value, close to 0.45, expectedly confirmed the diffusion of drug molecules through the HPMC matrix as the predominant mechanism with a limited share of erosion. This finding is in agreement with the literature data reporting that the very soluble drug is primarily released by the diffusion of the dissolved molecule of API through the hydrated gel layer of hydrophilic swelling polymers [62], including hypromellose matrix systems [63–66]. The addition of insoluble Eudragit® polymers led to an increase in the release exponent* n* for all Eudragit® samples (0.4756-0.7610) and supported the rising role of matrix erosion in a concentration-dependent manner as can be seen in Figure 3,* arrow n(KPM*). This finding was also supported by the fact that the determination coefficient R^2^ for Hixson-Crowell model (R^2^ > 0.98), except for NM samples, increased with a rising Eudragit® concentration in matrix. The presence of diffusion as a predominant mechanism of the API release was also confirmed by the good correspondence of the data with Higuchi (R^2^ > 0.99) and Baker-Lonsdale model (R^2^ > 0.98).

### 3.5. Texture Profiling, Gel Layer Dynamics of Swollen Matrices, and Characterization of Gel Layer by Cryo-SEM

**Figure 4:**
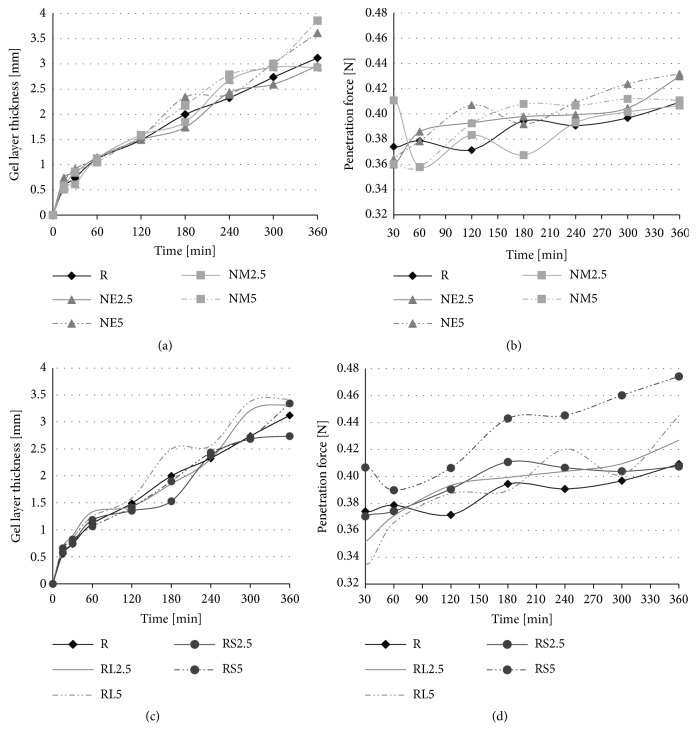
Gel layer thickness and the penetration force through the gel layer of tested samples during 360 minutes: (a), (c) Set 2.5: SD_max_ 0.58 mm; Set 5: SD_max_ 0.31 mm; (b), (d) Set 2.5: SD_max_ 0.02 N; Set 5: SD_max_ 0.04 N.

Drug release from hydrophilic matrices is controlled by the thickness and consistency of the gel layer created on the surface influencing both the* burst effect* at the beginning of the dissolution test and the overall dissolution profile [67]. Gel layer formation and its dynamic as a function of time (gel layer thickness and the rigidity of the gel) was measured by means of texture profiling analysis [43, 67, 68]. The gel layer thickness and penetration force through the gel layer are represented as a function of time in Figure 4.

**Figure 5:**
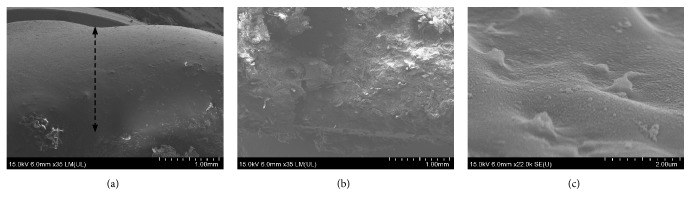
Surface gel layers of the matrix formulation NM2.5 by the cryo-SEM technique during the dissolution test with pH 6 after 3 hours: (a) cross-section of surface gel layer (the gel layer marked with an arrow), (b) nonhydrated core of the tablet, and (c) detail of the surface of a gel layer.

To verify the accuracy of the measurements, the hydrated tablet of the NM2.5 sample was cross-sectioned and observed by the cryo-SEM technique after 3 hours of the dissolution test (Figure 5(a)). The thickness of the gel layer was in good accordance with the texture profile measurement (Figure 4(a)). Moreover, cryo-scanning electron microscopic images of the nonhydrated core and the surface of the gel layer were obtained (Figures 5(b) and 5(c)). As it is demonstrated in Figures 4(a) and 4(c), the forming gel layer thickness was not significantly influenced by the Eudragit® present in the studied formulations. The result is quite different from the results obtained by Naiserova et al., where the presence of the insoluble Eudragit® polymers significantly reduced the thickness of the gel layer of the HPMC K4M/Eudragit® matrices at the beginning of the dissolution test [31]. An explanation could be seen in the use of the higher viscosity type of HPMC (K100M), which probably reduced the influence of Eudragit® polymers on swelling properties of HPMC [69]. However, the gel layer of Eudragit® samples exhibited higher values of penetration force necessary for its overcoming (see the penetration force Figures 4(b) and 4(d)). The creation of more rigid gel layers in comparison to the reference sample was probably responsible for the dissolution behavior of Eudragit® samples. This finding was supported by PCA where the negative correlation of the penetration force (see Figure 3(b)*, arrow force 300 min)* and dissolution characteristic at 300 and 720 minutes (see Figure 3(b),* arrow dis 300 min; dis 720 min)* can be observed. The penetration force also exhibited concentration-dependent behavior and rose with the increasing Eudragit® amount in tablets. These findings were positively confirmed by the results from ANOVA (p < 0.001). As it can be noticed, the reference sample R showed a more continuous course of gel layer thickness and the penetration force in time, whereas the course of these parameters fluctuated for Eudragit® formulations. An explanation could be seen in a more extensive erosion process in samples containing a certain proportion of insoluble matter. This could be responsible for some step changes in the internal structure of the forming gel layer. This result is in agreement with our previous study. On the other hand, the step changes in these characteristics were not as significant as when the low viscosity type HPMC K4M was used as a matrix carrier [31].

## 4. Conclusion

The application of an insoluble type of Eudragit® NE, NM, RL, and RS in the form of water dispersions directly on a very soluble drug levetiracetam during the high-shear granulation was feasible only in a multistep granulation process. It was demonstrated that not only the chemical structure of the used insoluble polymers played a role in dissolution behavior of prepared formulations. A key parameter could be seen also in the presence of surfactants in the used water dispersions. From this point of view, the adjustment of the dissolution profile of very soluble drug towards zero-order kinetic (lower* burst effect* and maximum of totally released API) was achievable only from HPMC K100M matrices containing the drug previously granulated by 30% water dispersion of Eudragit® NE containing a relatively high amount of surfactant nonoxynol 100 (1.5%).

## Figures and Tables

**Table 1:** Composition of granules and the number of granulation steps.

*Sample∗*	*Drug (g)*	*Eudragit® 30D dispersion (g)*				*No. of granulation steps*
		NE (g)	NM (g)	RL (g)	RS (g)	
*NE2.5 *	100.0	27.7	-	-	-	3
*NE5*	100.0	55.7	-	-	-	6
*NM2.5*	100.0	-	27.7	-	-	3
*NM5*	100.0	-	55.7	-	-	6
*RL2.5*	100.0	-	-	27.7	-	3
*RL5*	100.0	-	-	55.7	-	6
*RS2.5*	100.0	-	-	-	27.7	3
*RS5*	100.0	-	-	-	55.7	6

*∗*2.5 and 5.0 mean the amount of solid Eudragit® in the final tablets.

**Table 2:** Composition of matrix tablets.

*Sample*	*Drug*		*HPMC K100M*		*MCC PH 102*		*Eudragit®*	
	*(mg)*	(%)	*(mg)*	(%)	*(mg)*	(%)	*(mg)*	(%)
*R* ^*d*^	100.0	30	216.7	65	16.7	5.0	-	-
*NE2.5*	100.0	30	216.7 ^*ex*^	65	8.4 ^*ex*^	2.5	8.3	2.5
*NE5*	100.0	30	216.7 ^*ex*^	65	-	-	16.7	5.0
*NM2.5*	100.0	30	216.7 ^*ex*^	65	8.4 ^*ex*^	2.5	8.3	2.5
*NM5*	100.0	30	216.7 ^*ex*^	65	-	-	16.7	5.0
*RL2.5*	100.0	30	216.7 ^*ex*^	65	8.4 ^*ex*^	2.5	8.3	2.5
*RL5*	100.0	30	216.7 ^*ex*^	65	-	-	16.7	5.0
*RS2.5*	100.0	30	216.7 ^*ex*^	65	8.4 ^*ex*^	2.5	8.3	2.5
*RS5*	100.0	30	216.7 ^*ex*^	65	-	-	16.7	5.0

^ex^Extragranular excipients;  ^d^direct compression; each sample contains 0.5% of Aerosil® 200^ex^ and 2.5% of magnesium stearate ^ex^  for better flowability and compression feasibility.

**Table 3:** Flow properties of final mixtures and characteristics of matrix tablets.

*Sample*	*Hausner ratio*	*Flow evaluation*	*Friability*	*Hardness*	*Average drug content*	*Average weight*
	±* SD*		*[*%*]*	*[N] *±* SD*	*[*%*] *±* SD*	*[mg] *±* SD*
*R*	1.32 ± 0.01	Passable	0.15	81.6 ± 2.00	95.95 ± 1.02	338.7 ± 0.0018
*NE2.5 *	1.27 ± 0.02	Passable	0.13	92.1 ± 1.60	100.58 ± 5.34	345.3 ± 0.0028
*NE5*	1.24 ± 0.01	Fair	0.06	89.2 ± 1.80	104.84 ± 6.23	347.2 ± 0.0029
*NM2.5*	1.30 ± 0.00	Passable	0.10	86.3 ± 2.30	103.57 ± 2.01	340.3 ± 0.0025
*NM5*	1.25 ± 0.01	Fair	0.38	87.7 ± 2.30	105.54 ± 3.60	338.9 ± 0.0028
*RL2.5*	1.30 ± 0.02	Passable	0.08	92.7 ± 2.80	103.98 ± 1.12	341.7 ± 0.0033
*RL5*	1.22 ± 0.00	Fair	0.06	94.6 ± 2.80	103.43 ± 6.07	339.4 ± 0.0040
*RS2.5*	1.30 ± 0.01	Passable	0.10	96.8 ± 4.80	103.97 ± 1.45	345.4 ± 0.0034
*RS5*	1.30 ± 0.00	Passable	0.06	88.0 ± 3.60	106.56 ± 3.81	345.5 ± 0.0046

**Table 4:** Important characteristics of dissolution profiles and similarity factor analysis.

*Sample*	*Average burst effect in 30 min*	*Total released drug amount*	*Similarity factor analysis f*_*2*_	
	±* SD (*%)		*to the R*	*compared sample*
*R*	22.47 ± 0.32	100.58 ± 1.48	-	
*NE2.5*	16.08 ± 1.07	89.32 ± 2.47	59.74	*NE2.5 / NE5* 71.02
*NE5*	8.04 ± 0.43	97.02 ± 1.95	52.55	
*NM2.5*	20.23 ± 0.66	93.58 ± 1.56	71.03	*NM2.5 / NM5* 75.52
*NM5*	17.18 ± 0.62	84.75 ± 5.46	61.00	
*RL2.5*	15.86 ± 0.89	86.17 ± 3.69	75.05	*RL2.5 / RL5* 92.84
*RL5*	13.70 ± 0.46	89.57 ± 2.38	69.86	
*RS2.5*	14.94 ± 0.66	96.48 ± 2.43	72.38	*RS2.5 / RS5* 46.98
*RS5*	8.61 ± 0.95	77.47 ± 4.89	44.05	

**Table 5:** Mathematical modelling and drug release kinetics from matrices.

*Model*	*Higuchi*	*Korsmeyer-Peppas*		*Zero- order*	*First- order*	*Hixson-Crowell*	*Baker-Lonsdale*
*Sample*	R^2^	R^2^	n	R^2^	R^2^	R^2^	R^2^

*R*	0.9981	0.9989	0.4751	0.9023	0.9949	0.9934	0.9906
*NE2.5 *	0.9975	0.9982	0.5532	0.9228	0.9924	0.9902	0.9854
*NE5*	0.9989	0.9882	0.6670	0.9579	0.9949	0.9944	0.9774
*NM2.5*	0.9986	0.9990	0.4756	0.9162	0.9854	0.9930	0.9767
*NM5*	0.9965	0.9998	0.5273	0.9121	0.9979	0.9895	0.9970
*RL2.5*	0.9870	0.9960	0.6855	0.9124	0.9911	0.9771	0.9944
*RL5*	0.9885	0.9963	0.7610	0.9223	0.9945	0.9817	0.9943
*RS2.5*	0.9945	0.9962	0.6559	09248	0.9895	0.9892	0.9827
*RS5*	0.9976	0.9835	0.6997	0.9473	0.9947	0.9919	0.9800

## Data Availability

All data supporting the findings of this study are included within the article.
